# Response to ‘Nalmefene in alcohol‐dependent patients with a high drinking risk: A limited efficacy in reducing alcohol consumption’

**DOI:** 10.1111/pcn.12971

**Published:** 2020-01-25

**Authors:** Susumu Higuchi, Hisatsugu Miyata, Takako Hayashi

**Affiliations:** ^1^ National Hospital Organization Kurihama Medical and Addiction Center Yokosuka Japan; ^2^ Department of Psychiatry Jikei University School of Medicine Tokyo Japan; ^3^ Medical Affairs, Otsuka Pharmaceutical Co., Ltd. Tokyo Japan

Palpacuer and colleagues pointed out that Nalmefene for reducing alcohol consumption in alcohol‐dependent patients was authorized in Europe based only on subgroup analyses of ESENSE 1, 2 and SENSE studies.^1^ We agree with this concern and are glad that we were able to demonstrate the efficacy of Nalmefene in reducing alcohol consumption in alcohol‐dependent patients with a high or very high drinking risk level (DRL) *via* a prospective randomized controlled trial. As such, this is the first study to validate the efficacy of Nalmefene without the use of a post‐hoc analysis.

On the other hand, we can understand some of the concerns that Palpacuer and colleagues raised in their letter.

First, we fully understand the issue of attrition bias^2^ and had mentioned this as a limitation in our paper.^3^ We also performed two kinds of imputation analyses and it was shown that heavy drinking day (HDD) and total alcohol consumption (TAC), the main analyses, were robust with sensitivity analysis complementing missing data.^3^

The other point raised was the study period of 12 and 24 weeks. It should be noted that 12 or 24 weeks have generally been adopted as the evaluation period for alcohol dependence treatments in clinical trials^4^, ^5^, ^6^; however, we agree that 6 months is too short to evaluate efficacy for harm reduction.

In terms of the point on harm reduction, we value outcome measures including mortality or quality of life, and consider accident, injuries, and somatic alcohol‐related complications as crucial endpoints, which should be included in “harm reduction” with reducing alcohol intake.^7^

Quality of Life was evaluated in our study, and a significant difference was found between placebo and Nalmefene groups at 12 weeks for the Alcohol Quality of Life Scale (AQOLs) evaluation.^3^ However, as Palpacuer and colleagues pointed out, no significant differences were found between placebo and Nalmefene groups at 24 weeks for AQOLs, SF‐36 and EQ‐5D evaluations.^3^ As neither the number of patients nor the study plan had been designed for QOL evaluation in this study, another clinical study to evaluate crucial endpoints with appropriately designed population and study period is warranted.

Palpacuer and colleagues also mentioned that the clinical significance of a statistically significant difference on a surrogate outcome should be critically appraised. As discussed earlier, the goal for harm reduction should include many aspects, although we consider HDD and TAC as reliable endpoints to evaluate treatments for short‐term reduction in alcohol consumption. HDD counts the number of days with heavy drinking and TAC records how much alcohol is consumed by patients. These markers are surrogate for harm reduction, however, both of them directly measure alcohol consumption. Both HDD and TAC are recommended as primary endpoints of treatment for reducing alcohol consumption by the European Medicines Agency (EMA) guideline because “HDD are associated with specific risks such as acute cardiovascular outcomes or accidents”.^8^ It has also been reported that high volumes of drinking per occasion predicted negative social consequences independently of overall drinking volume.^9^ In addition, it was suggested that “Any reduction in WHO risk drinking level during treatment was associated with significantly fewer alcohol‐related consequences and improved mental health at the end of treatment and for up to 1 year post‐treatment.”^10^ These results suggest that HDD is a reliable marker that affects events of relatively short period. However, as we agree that both measures evaluate short‐term efficacy, long‐term or real‐world based studies need to include other markers to evaluate clinical significance of the treatment as discussed earlier.

We recognize the importance of long‐term psychosocial interventions by trained professionals, but in addition to them, it should be beneficial for patients to have multiple treatment choices, especially when they can use some interventions in combination.

## Disclosure statement

Dr. Higuchi reports personal fees from Otsuka pharma Co. Ltd., other from Otsuka Pharma Co. Ltd., grants from Otsuka Pharma Co. Ltd., grants from Lundbeck Japan, other from Lundbeck Japan, during the conduct of the study; personal fees from Nippon Shinyaku, personal fees from MSD, personal fees from Yoshitomi Pharmaceutical, personal fees from Jansen Pharma, personal fees from Eli‐Lilley Japan, personal fees from Mochida Pharmaceutical, personal fees from Meiji‐Seika Pharma, other from Nippon Shinyaku, other from Eisai, outside the submitted work; Dr. Miyata reports personal fees from Otsuka Pharmaceutical Co., Ltd., during the conduct of the study; personal fees from Otsuka Pharma Co., Ltd., personal fees from Meiji Seika Pharma Co., Ltd., personal fees from Janssen Pharmaceutical K.K., personal fees from Sumitomo Dainippon Pharma Co., Ltd., personal fees from Mochida Pharmaceutical Co., Ltd., personal fees from Eli Lilly Japan K.K., grants from Smoking Research Foundation, grants from Japan Tobacco Inc., grants from Suntory Global Innovation Center, outside the submitted work; Takako Hayashi are full‐time employees of Otsuka Pharma Co., Ltd. A California winery, Ridge Vineyards, Inc., is a subsidiary company of Otsuka Pharma Co. Ltd.

## References

[1] Palpacuer C , Braillon A , Naudet F . Nalmefene in alcohol‐dependent patients with a high drinking risk: A limited efficacy in reducing alcohol consumption. Psychiatry Clin. Neurosci. 2020; 74: 218. 31642148 10.1111/pcn.12946

[2] Dumville JC , Torgerson DJ , Hewitt CE . Reporting attrition in randomised controlled trials. BMJ 2006; 332: 969–971. 16627519 10.1136/bmj.332.7547.969 PMC1444839

[3] Miyata H , Takahashi M , Murai Y *et al* Nalmefene in alcohol‐dependent patients with a high drinking risk: A randomized controlled trial. Psychiatry Clin. Neurosci. 2019; 73: 697–706. 31298784 10.1111/pcn.12914 PMC6899457

[4] Higuchi S . Efficacy of accamprosate for the treatment of alcohol dependence long after recovery from withdrawal syndrome: A randomized, double‐blind, placebo‐controlled study conducted in Japan (sunrise study). J. Clin. Psychiatry 2015; 76: 181–188. 25742205 10.4088/JCP.13m08940

[5] Reynaud M , Aubine h‐J , Trinquet F , Zakine B *et al* A randomized, placebo‐controlled study of high‐dose baclofen in alcohol‐dependent patients‐the ALPADIR study. Alcohol Alcohol. 2017; 52: 439–446. 28525555 10.1093/alcalc/agx030

[6] Petrakis IL , Ralevski E , Gueorguieva R , O'Malley SS , Arias A *et al* Mecamylamine treatment for alcohol dependence: A randomized cotrolled trial. Addiction 2017; 113: 6–14. 28710873 10.1111/add.13943 PMC5725262

[7] Naudet F , Palpacuer C , Boussageon R , Laviolle B . Evaluation in alcohol use disorders ‐ insights from the nalmefene experience. BMC Med. 2016; 14: 119. 27534932 10.1186/s12916-016-0664-9 PMC4989362

[8] European Medicines Agency Committee for Medicinal Products for Human use (CHMP) . Guideline on the development of medicinal products for the treatment of alcohol dependence [Internet]. European Medicines Agency; 2010 [Cited 15 November 2019.] Available from URL: http://www.ema.europa.eu/docs/en_GB/document_library/Scienti?c_guideline/2010/03/WC500074898.pdf

[9] Rehm J , Gmel G . Patterns of alcohol consumption and social consequences. Results from an 8‐year follow‐up study in Switzerland. Addiction 1999; 94: 899–912. 10665078 10.1046/j.1360-0443.1999.94689912.x

[10] Witkiewitz K , Hallgren KA , Kranzler HR *et al* Clinical validation of reduced alcohol consumption after treatment for alcohol dependence using the World Health Organization risk drinking levels. Alcohol. Clin. Exp. Res. 2017; 41: 179–186. 28019652 10.1111/acer.13272 PMC5205540

